# Perceived parental emotional warmth and academic burnout in physical education pre-service teachers: the chain mediating roles of physical activity and smartphone addiction

**DOI:** 10.3389/fpsyg.2026.1853224

**Published:** 2026-08-11

**Authors:** Shujia Liu, Tong Liu, Hui Ma, Zhonggen Yin, Chen Zhu, Wenjie Cheng

**Affiliations:** 1 Geely University of China, College of Physical Education and Health, Chengdu, China; 2 Chengdu Sport University, School of Sports Training, Chengdu, Sichuan, China; 3 Chengdu Sport University, School of Marxism, Chengdu, Sichuan, China; 4 Chongqing University of Education, College of Physical Education and Health Management, Chongqing, China; 5 Chongqing Three Gorges University of Science and Technology, College of Physical Education and Health, Chongqing, China; 6 Sichuan Fine Arts Institute, School of General Education, Chongqing, China

**Keywords:** academic burnout, chain mediating, perceived parental emotional warmth, physical activity, smartphone addiction

## Abstract

**Objective:**

Academic burnout is a prominent challenge for physical education pre-service teachers facing dual pressures of academic study and physical training. This study aimed to examine the statistical association between perceived parental emotional warmth and academic burnout. Guided by self-determination theory and the behavioral substitution perspective, we also explored the chain mediating pathways involving physical activity and smartphone addiction to provide an integrated theoretical framework.

**Methods:**

A cross-sectional design was adopted. In December 2025, a stratified sample of 1,192 physical education pre-service teachers from 15 Chinese provinces completed the S-EMBU (perceived parental emotional warmth), Physical Activity Scale, SAS-SV, and ALBI. Data were analyzed using PROCESS Model 6 in SPSS 25.0 with 5,000 bootstrap resamples.

**Results:**

Correlation analysis showed all variables significantly intercorrelated. After controlling for gender and grade, the chain mediation model was supported. Perceived parental emotional warmth showed a direct negative statistical association with academic burnout and was also linked via three indirect pathways: (1) physical activity alone (β = −0.290, 95% CI [−0.323, −0.257]); (2) smartphone addiction alone (β = −0.026, 95% CI [−0.040, −0.012]); and (3) the sequential pathway via physical activity then smartphone addiction (β = −0.066, 95% CI [−0.080, −0.054]).

**Conclusion:**

Perceived parental emotional warmth is associated with lower academic burnout among physical education pre-service teachers, partially through a behavioral sequence of increased physical activity and reduced smartphone addiction. Due to the cross-sectional design, causal inferences cannot be drawn. Nevertheless, these findings suggest perceived parental emotional warmth may associate with academic outcomes through a dynamic process associated with constructive behaviors and reduced risky behaviors. The results provide a novel behavioral-ecological perspective and may inform the development of future interventions combining family support, health behavior promotion, and digital literacy training.

## 1 Introduction

Academic burnout is a state of emotional exhaustion, cynicism, and reduced personal accomplishment that results from prolonged academic stress (Wang et al., 2024b). It has become an important factor affecting university students’ academic performance and mental health. Academic burnout is not only closely related to low study engagement (Olson et al., 2025) and increased procrastination (Alexander Govicar et al., 2024), but may also trigger persistent anxiety and depression (Sun et al., 2024), thereby harming students’ long-term career development potential (Mohebbi et al., 2021). For physical education pre-service teachers, the issue of academic burnout is particularly noteworthy. These students must not only complete demanding coursework in educational theory and professional skills but also maintain high-intensity physical training. This dual load of cognitive and physical effort exposes them to unique risks of physical and mental exhaustion (Öztürk Çelik, 2025). Moreover, for these future teachers, physical activity is not merely a current health behavior but may reflect a developmental trajectory of sustained sport participation from childhood through adolescence into adulthood (Daga et al., 2026). This life-course perspective suggests that their current physical activity habits are deeply embedded in their professional identity formation. The academic status of physical education pre-service teachers is not only a matter of personal growth, but also directly related to the quality of future primary and secondary school physical education and the foundation of youth health promotion.

Research has shown that positive parenting style in the family system, as the initial environment of individual development, has a stable association with academic burnout among university students (Yang et al., 2025). However, whether and how this association operates in the specific group of physical education pre-service teachers, especially through observable daily behavioral sequences, remains underexplored. This enduring influence is theoretically expected, as early family experiences shape internal working models and self-regulation patterns that remain active in young adulthood (Arnett, 2000).

In recent years, researchers have used mediation models to reveal the complex pathways linking perceived parental emotional warmth to child development. However, most existing studies have focused on the sequential transmission of internal psychological traits. For example, perceived parental emotional warmth is associated with higher self-esteem, which in turn is associated with lower academic procrastination and better self-regulated learning (Kim and Kim, 2021). Few studies have extended the perspective to explicit and competing daily behavioral chains. In the formation process of academic burnout, there is a logically plausible but empirically untested behavioral sequence: the family rearing environment first shapes the individual’s basic health behavior patterns, such as physical activity, and this pattern is then associated with risk behavior choices when coping with stress and leisure, such as smartphone use. Together, these two behaviors constitute a behavioral pathway that influences academic psychological states.

This hypothesis is grounded in the integration of two theoretical frameworks. Self-determination theory (Ryan and Deci, 2000) suggests that a supportive family environment satisfies individuals’ basic psychological needs (autonomy, competence, relatedness), which is associated with intrinsically motivated health behaviors such as regular physical activity (Rangriz et al., 2025; Lev Arey et al., 2022). Conversely, when these needs are frustrated, as may occur in less supportive family environments, individuals may turn to immediately rewarding but maladaptive behaviors, which may be associated with an increased risk of smartphone addiction (Zhang et al., 2022).

The behavioral substitution perspective further posits that different behaviors compete for limited time and psychological resources. Two interrelated mechanisms are involved: resource competition, meaning that physical activity is associated with fewer opportunities for smartphone use; and functional substitution, meaning that the positive psychological experiences derived from physical activity may be associated with lower motivation for smartphone addiction (Zhu et al., 2025, Ye et al., 2025). The integration of these two perspectives represents a complete theoretical chain from family environment to health behavior, to risk behavior, and finally to academic outcomes.

**FIGURE 1:**
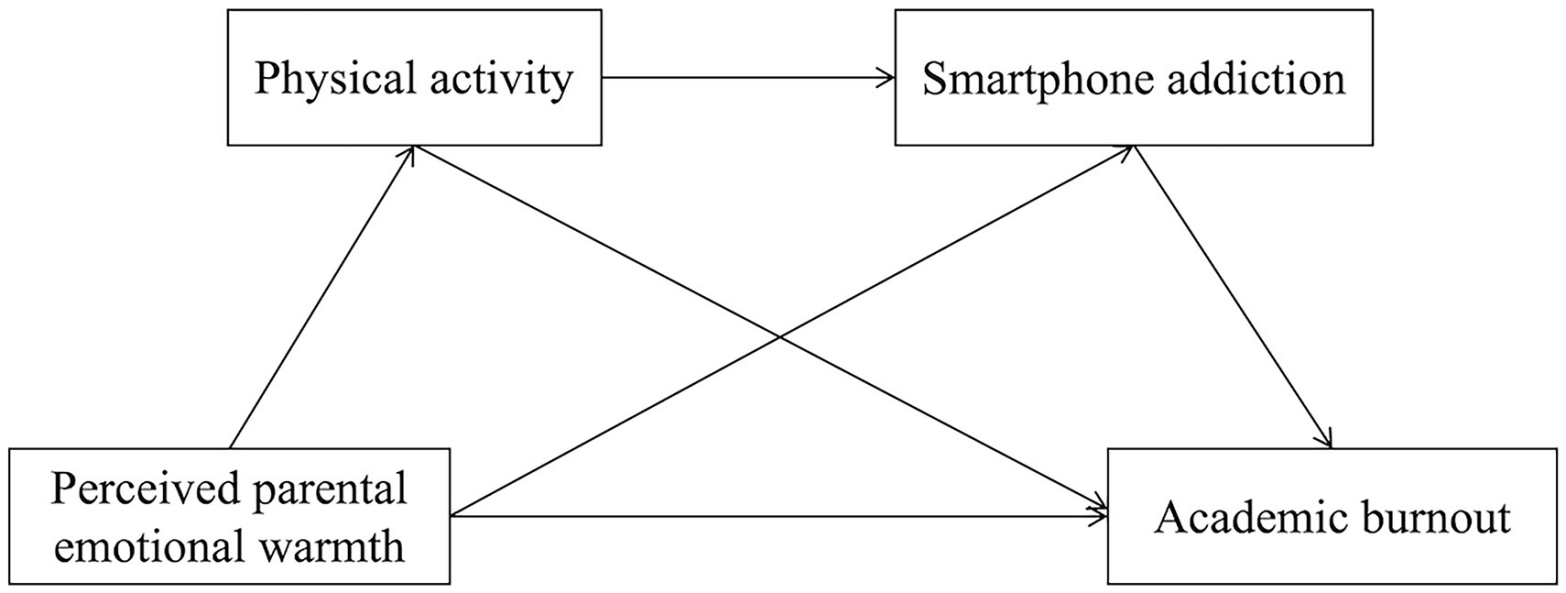
The chain mediation effect model.

Therefore, to explore the potential mechanisms between perceived parental emotional warmth and academic burnout among physical education pre-service teachers, this study integrates self-determination theory and the behavioral substitution perspective to propose a chain mediation model. We hypothesize that the association between perceived parental emotional warmth and academic burnout is statistically consistent with a chain mediation model involving physical activity and smartphone addiction (see Figure 1). Specifically, we propose three indirect pathways: (1) via physical activity alone, (2) via smartphone addiction alone, and (3) sequentially via physical activity followed by smartphone addiction.

Despite the theoretical plausibility of this chain model, empirical evidence specifically examining these sequential mediating roles among physical education pre-service teachers remains limited. The present study adopts a cross-sectional design to provide a preliminary test of the above theoretical model. The findings may offer a more integrated behavioral-ecological framework for understanding the correlates of academic burnout, from family environment to health behavior, to risk behavior, and finally to academic outcomes, and may inform future school-family collaborative support strategies.

## 2 Literature review and hypothesis development

### 2.1 Perceived parental emotional warmth and academic burnout

Perceived parental emotional warmth refers to the earliest and most enduring micro-environmental system in an individual’s development. It describes a relatively stable set of behavioral tendencies and emotional attitudes that parents display when raising their children. In empirical research, this construct is often operationalized using key dimensions such as emotional warmth and understanding versus overprotection and rejection. Higher scores indicate higher levels of positive parenting characteristics (Luo et al., 2023). Academic burnout is a psychological syndrome characterized by emotional exhaustion, cynicism toward learning, and reduced personal accomplishment, resulting from prolonged academic stress (Andargeery et al., 2025). Developmental ecological theory points out that the family, as a core “microsystem,” is associated with the individual’s adaptation and development outcomes (Bronfenbrenner, 2013). A large body of research has confirmed that the overall characteristics of positive parenting style are stably associated with the level of academic burnout among adolescents and university students (Ding et al., 2019; Guo et al., 2024).

Specifically, higher scores on perceived parental emotional warmth typically reflect a more supportive, warm, and understanding family interaction climate. This climate provides children with crucial emotional resources and space for autonomous development, helps them form intrinsic academic motivation, effective stress coping strategies, and greater psychological resilience, thereby serving as an important protective factor against academic burnout (Strage and Brandt, 1999). Conversely, lower scores on positive parenting style may be associated with a family environment characterized by insufficient support, excessive interference, or emotional coldness. Such environments may weaken children’s autonomy, increase their experience of stress, or directly deprive them of emotional support, thus making them more prone to emotional exhaustion, academic cynicism, and other burnout manifestations (Yang et al., 2025).

Although ample evidence has accumulated from studies of general university student populations, direct investigations of the relationship between perceived parental emotional warmth and academic burnout remain relatively limited among physical education pre-service teachers, a group that bears the dual roles of academic pressure (as teacher students) and training load (as sports students). The need for family support and autonomous space may be more pronounced in this group due to the specific demands of their professional training, such as self-discipline, perseverance, and physical performance. This suggests that the pathways through which the family environment affects their academic status may be unique.

Based on this, we propose the following baseline hypothesis:

H1: Perceived parental emotional warmth is negatively associated with the level of academic burnout.

### 2.2 Perceived parental emotional warmth, physical activity, academic burnout

This section aims to explore the potential mediating role of physical activity in the association between perceived parental emotional warmth and academic burnout, thereby constructing the first theoretical pathway: “family environment → health behavior → academic adaptation.”

First, perceived parental emotional warmth is a key family antecedent that shapes an individual’s physical activity behavior (Yao and Rhodes, 2015). According to self-determination theory, when parents provide emotional warmth, autonomy support, and appropriate challenges, this pattern is associated with greater satisfaction of the three basic psychological needs of children: autonomy, competence, and relatedness (Guo et al., 2021). This state of need satisfaction directs individuals toward more intrinsically motivated activities, and physical activity is a typical example of such activities. Research has shown that a supportive family physical activity environment can satisfy adolescents’ needs for autonomous choice, skill improvement, and social connection, thereby promoting the internalization of exercise motivation and forming more persistent and deeper exercise motivation (Jiang and Xiao, 2024). In contrast, lower scores on positive parenting style may be associated with experiences such as insufficient support and excessive control. High levels of parental control reduce adolescents’ perception that their actions originate from their own decisions, which is typically associated with less physical activity practice (Vega-Díaz et al., 2023). For physical education pre-service teachers, physical activity is not only a general health behavior but also the core of their professional skill development. Therefore, the overall level of family support may have a particularly profound association with their ability to maintain regular training.

Second, regular physical activity is statistically associated with lower levels of academic burnout, consistent with a protective role (Tian and Yang, 2024). Its mechanisms can be understood at both physiological and psychological levels. Physiologically, physical activity improves cerebral oxygenation and promotes the secretion of neurotransmitters such as endorphins, directly alleviating the physical tension and fatigue caused by stress (Caen et al., 2019). Psychologically and socially, physical activity is a positive process of restoring and rebuilding an individual’s psychological resources (Guillemin et al., 1977). First, it provides an emotional outlet for academic stress (Nawaz et al., 2025). Second, the skill improvement and goal achievement obtained during exercise can effectively enhance self-efficacy and a sense of accomplishment, which directly counteracts the reduced personal accomplishment dimension of academic burnout (Bandura, 1977, Zetou et al., 2012). Third, team-based sports activities can also provide valuable social support (Kim et al., 2023).

In summary, perceived parental emotional warmth may be indirectly associated with the level of academic burnout among physical education pre-service teachers by influencing their participation in regular physical activity. Based on this, the study proposes the following hypothesis.

H2: Physical activity mediates the relationship between perceived parental emotional warmth and academic burnout.

### 2.3 Perceived parental emotional warmth, smartphone addiction, and academic burnout

After discussing the potential mediating role of the positive behavior (physical activity), this section turns to examine smartphone addiction as a negative risk behavior in the digital age. It aims to construct a second explanatory pathway linking perceived parental emotional warmth to academic burnout. This pathway focuses on how, when individuals’ basic psychological needs are frustrated in the family environment, they may turn to the virtual world for compensation, and how this compensatory satisfaction may deplete the psychological resources needed for academic adaptation.

As an antecedent variable of smartphone addiction, the explanatory mechanism of perceived parental emotional warmth is based on the need compensation model. Drawing on self-determination theory, when individuals perceive low levels of perceived parental emotional warmth, this often means that their basic psychological needs such as autonomy and relatedness are not sufficiently satisfied in family interactions (Li et al., 2025). To compensate for this relative lack of basic psychological needs in reality, individuals may tend to immerse themselves deeply in the virtual environment constructed by smartphones (Chen et al., 2025). In the virtual environment, individuals can relatively easily obtain immediate feedback, such as likes from social media or rewards from games, experience a sense of control over information browsing and interaction, and establish low threshold social connections. In this way, they form a compensatory experience for unmet psychological needs (Chen et al., 2025). Existing empirical studies support this view: parental emotional warmth is negatively correlated with adolescent smartphone addiction, whereas parental rejection and overprotection are positively correlated with addiction (Yogesh et al., 2024). For physical education pre-service teachers, this mechanism may be particularly noteworthy. They simultaneously bear the dual pressures of academic study and physical training. If they perceive low levels of perceived parental emotional warmth, they may be more likely to use smartphones as a convenient way to regulate emotions and as a low stress social channel, thereby increasing the risk of developing maladaptive usage patterns.

Smartphone addiction is a clear risk factor that exacerbates academic burnout. Its mechanisms mainly involve resource depletion (Wang et al., 2025b) and cognitive interference (Al-Abyadh et al., 2024). First, according to the limited self-control theory, excessive and uncontrollable use of smartphones continuously consumes an individual’s cognitive resources and self-regulatory capacity (Viacava et al., 2016). As a result, when facing academic tasks that require sustained effort, the psychological energy needed to maintain concentration and exert volitional control is more likely to be depleted prematurely. Second, this behavioral pattern is often accompanied by a significant decline in sleep quality, substantial fragmentation of study time, and scattered deep attention, directly impairing learning efficiency and knowledge integration (Amin and Sohail, 2025). Finally, excessive engagement in online interactions may crowd out more supportive and deeper social opportunities in real life, which may in turn exacerbate feelings of alienation and loneliness in academic contexts (Kraut et al., 1998).

Therefore, perceived parental emotional warmth may be indirectly associated with the level of academic burnout among physical education pre-service teachers by predicting their tendency toward smartphone addiction. Specifically, lower scores on positive parenting style may be associated with a higher risk of smartphone addiction, and this addictive behavior (Wang et al., 2024a), as a continuous process of psychological resource depletion, systematically erodes an individual’s time management ability, cognitive focus, and emotional balance, ultimately relating to more severe academic burnout (Yao et al., 2025). Conversely, higher levels of perceived parental emotional warmth may be associated with lower motivation for virtual compensation, and this greater satisfaction of basic psychological needs may be associated with a buffering effect on the risk pathway.

Thus, we propose the following hypothesis:

H3: Smartphone addiction mediates the relationship between perceived parental emotional warmth and academic burnout.

### 2.4 Perceived parental emotional warmth, physical activity, smartphone addiction, and academic burnout

The previous sections discussed the independent mediating roles of physical activity and smartphone addiction in the relationship between perceived parental emotional warmth and academic burnout. However, from a theoretical perspective, these two daily behavioral patterns are not isolated from each other. Instead, they are more likely to form a sequential transmission process with an internal logical order. When explaining the theoretical basis of this chain pathway, it is necessary to first clarify the connotation of the “behavioral substitution perspective.” This perspective originates from the limited resource assumption in behavioral economics and self-control theory. Its core logic is that an individual’s time, attention, and self-control resources are limited. An increase in one activity necessarily reduces the resources available for another activity, thereby creating a “substitution” relationship between behaviors (Precht et al., 2024). Unlike self-determination theory, which focuses on how the satisfaction or frustration of psychological needs affects behavioral motivation, the behavioral substitution perspective focuses on the dynamic competitive relationship between behaviors. That is, once a constructive behavior (such as physical activity) occupies resources, the space for risky behaviors (such as excessive smartphone use) is naturally compressed. The integration of these two perspectives constitutes the theoretical framework of this study. Self-determination theory explains the starting point of the chain: how parenting initiates health behaviors through need satisfaction. The behavioral substitution perspective explains the middle link: how physical activity inhibits smartphone addiction through resource competition, thereby affecting academic burnout. This integration avoids simply juxtaposing the two perspectives and instead forms a complete explanatory chain of “motivation → behavioral competition → adaptation outcomes.”

First, theoretical reasoning supports a dynamic trade off relationship between physical activity and smartphone use. This conclusion is based on the dual perspectives of resource competition and functional substitution (Zhu et al., 2025). Resource competition, grounded in the limited resource assumption, holds that an individual’s time, attention, and psychological energy are finite. When an individual engages in high intensity physical activity, the resources available for passive screen behaviors (such as smartphone use) necessarily decrease, thereby objectively reducing the possibility of excessive smartphone use (Precht et al., 2024). This is a quantitative competition, emphasizing a zero-sum game in resource allocation. Behavioral substitution focuses on the compensatory relationship at the functional level. Its core logic is that if one behavior can provide the psychological satisfaction sought by another behavior (such as autonomy, competence, positive emotions), the former can substitute for the function of the latter, thereby reducing the individual’s motivational dependence on the latter. For example, regular physical activity provides immediate feelings of accomplishment, emotional release, and social connection. These psychological benefits can substitute for the virtual rewards and escapist compensation provided by smartphones, thereby actively reducing addiction tendency, rather than merely passively occupying time (Gerber et al., 2025).

In the theoretical model of this study, resource competition explains “how physical activity reduces the objective opportunity for smartphone use” (a necessary condition for the indirect pathway). Behavioral substitution explains “why physical activity can reduce the psychological motivation for smartphone addiction” (a direct inhibitory mechanism). Together, they form the theoretical basis for the “physical activity → smartphone addiction” link, but at different levels: resource competition is an objective behavioral constraint, while behavioral substitution is a psychological motivational shift. Clearly distinguishing between the two helps avoid theoretical confusion and makes the interpretation of subsequent mediation effects more precise. Existing empirical studies have provided preliminary evidence showing a stable negative relationship between physical activity level and the tendency for excessive smartphone use (Ye et al., 2025, Barot and Patel, 2023, Pirwani and Szabo, 2025).

Second, this behavioral sequence provides a more complete explanatory framework for understanding the long-term effects of perceived parental emotional warmth. According to self-determination theory, a higher level of positive parenting style is generally associated with an environment that better satisfies an individual’s basic psychological needs (Ryan and Deci, 2000). This helps promote the formation and maintenance of regular physical activity habits, constituting the starting point of the behavioral sequence (Tapia-Serrano et al., 2023). Subsequently, the physical and mental resources and positive feedback obtained through regular exercise not only produce direct benefits but may also inhibit the tendency to develop smartphone addiction through the dual mechanisms of resource competition and need satisfaction. Ultimately, this continuous process from gaining constructive behavior to reducing risky behavior reserves more abundant resources for individuals to cope with academic stress, thereby forming an effective buffer against academic burnout. Conversely, a lower level of perceived parental emotional warmth may be associated with a different behavioral pattern: lower participation in physical activity, leading to insufficient gain of psychological resources, while the individual may be more inclined to turn to smartphones for immediate need compensation, which may in turn exacerbate the occurrence of academic burnout.

Although existing studies have begun to use chain mediation models to explore the pathways of positive parenting style, and some have focused on behavioral variables (Li et al., 2023), research that systematically examines the chain mediating role of physical activity and smartphone addiction as an internally competitive behavioral sequence in the relationship between perceived parental emotional warmth and academic burnout remains rare, especially among the specific group of physical education pre-service teachers. For physical education pre-service teachers, physical activity is not only a health habit but also a core component of their professional practice. Smartphone addiction represents a typical self-regulation risk in digital life. These two behaviors may form a mutually constraining relationship in an individual’s time allocation and psychological resource management, thereby constituting a behavior transmission pathway with group specificity. Therefore, testing the mediating role of this specific behavioral sequence is significant not only for methodological application but also for empirically testing a clear theoretical proposition: whether constructive health behaviors can inhibit risky digital behaviors and thereby affect academic adaptation outcomes. This helps move research from exploring isolated psychological or behavioral factors to focusing on how the combination and sequence of daily behavioral patterns transmit environmental influences, thus providing a more integrated behavioral ecological perspective for understanding how the family environment shapes academic status.

Therefore, we propose the following hypothesis:

H4: Physical activity and smartphone addiction play a chain mediating role between perceived parental emotional warmth and academic burnout (perceived parental emotional warmth → physical activity → smartphone addiction → academic burnout) (See Figure 1. The chain mediation effect model).

## 3 Methodology

### 3.1 Procedures and participants

Sampling design. A stratified cluster sampling method was used. The stratification variables were geographical region (eastern, central, western, southern, and northern) and school type (schools of physical education within normal universities, and physical education departments within comprehensive universities). Within each geographical region, three provinces were randomly selected according to the list of higher education institutions published by the Ministry of Education, resulting in a total of 15 provinces. Within each selected province, one university that met the school type criteria was randomly selected. The random selection procedure was implemented using a random number generator. After selection, the head of the physical education department at each university was contacted to explain the study purpose and invite participation. All contacted universities agreed to participate, no replacement was required. The final sample covered 15 universities across 15 provinces in China. The sample size varied across universities, reflecting the natural variation in the size of physical education departments across institutions. While detailed per-institution participant numbers were not systematically recorded, the total sample (*N* = 1,192) and the broad geographic coverage (15 provinces) provide a reasonable basis for evaluating the representativeness of the sample. In each selected university, undergraduate students majoring in physical education or sports training from the first to the fourth year were sampled at the class level.

Sample size planning. *A priori* power analysis was conducted using G*Power 3.1 software. The parameters were set as follows: effect size f^2^ = 0.15 (medium effect), α = 0.05, and statistical power 1−β = 0.80. The minimum required sample size for a linear regression model was 107. It should be noted that this initial calculation was based on a simplified linear regression model. However, the final sample of 1,192 participants substantially exceeds the widely recommended 5–10 participants per scale item for factor analysis (Tinsley and Tinsley, 1987) which would require 180–360 participants for the 36 items in this study. Considering the complexity of the chain mediation model and the possibility of invalid questionnaires, we planned to recruit at least 1,000 participants. A total of 1,300 questionnaires were distributed.

Data collection procedure. Trained physical education teachers served as examiners and organized the questionnaire administration in class during regular classroom time. Participants scanned a QR code using their personal smartphones to enter the online survey platform^1^ and completed the questionnaires independently in the classroom. The examiners supervised from the podium to ensure that participants did not discuss with each other or drop out midway, but they did not intervene in the content of the responses. This mode of “offline gathering and online answering” ensured a controlled environment (teacher supervision, unified timing) while taking advantage of the platform’s automatic logic skipping and data collection functions. The completion time was approximately 15 to 20 min.

Criteria for excluding invalid questionnaires. After data collection, the questionnaires were screened one by one according to the following criteria: (1) completion time < 5 min or > 40 min (extreme values); (2) presence of obvious response patterns (e.g., selecting the same option for more than 10 consecutive items); (3) inconsistent answers to reverse coded items (e.g., contradictory responses); and (4) missing more than 2 pieces of basic information. Based on these criteria, a total of 108 invalid questionnaires were excluded. The effective sample size was 1,192, yielding an effective response rate of 91.69%.

Inclusion and exclusion criteria for participants. Inclusion criteria were: (1) full time undergraduate students majoring in physical education or sports training; (2) age between 18 and 24 years; and (3) voluntary participation with signed electronic informed consent. Exclusion criteria were: (1) having a serious physical illness (e.g., heart disease, unhealed bone fracture) that affected daily activities; (2) having a diagnosed mental illness (e.g., major depression, schizophrenia) currently in an active episode; and (3) having received systematic psychological treatment within the past three months. All participants were informed that they had the right to withdraw from the study at any time without any penalty.

Final sample composition. Participants ranged in age from 18 to 24 years (M = 19.5, SD = 1.265). There were 648 males (54.36%) and 544 females (45.64%). Grade distribution: first year, 471 (39.51%); second year, 342 (28.69%); third year, 266 (18.96%); fourth year, 113 (9.48%). Household registration type: urban, 580 (48.66%); rural, 612 (51.34%).

### 3.2 Ethics approval and consent to participate

This study strictly followed the ethical guidelines of the Declaration of Helsinki. The research protocol was approved by the Ethics Committee of Chengdu Sport University (approval number: CTYLL2025235). Before formal participation, all participants signed an electronic informed consent form through the online platform. The informed consent form detailed the study purpose, voluntary participation principle, confidentiality agreement, and the right to withdraw. After data collection, all information was anonymized. The anonymized dataset will be stored in accordance with the institutional ethical guidelines and data management policy and will be available for research use for at least five years after publication. Qualified researchers may request access to the data but must sign a formal data sharing agreement to ensure the privacy of participants and comply with the original informed consent terms.

### 3.3 Measurement tools

#### 3.3.1 Perceived parental emotional warmth scale

Positive parenting style was measured using the Emotional Warmth subscale of the short form of the Egna Minnen av Barndoms Uppfostran (S-EMBU) developed by Arrindell et al. (1999). The full S-EMBU has 21 items across three dimensions: Rejection (6 items, e.g., “My parents often lost their temper with me for no reason”), Emotional Warmth (7 items, e.g., “My parents often praised me”), and Overprotection (8 items, e.g., “I wish my parents would not worry too much about what I am doing”). In this study, the Emotional Warmth subscale was used to operationalize positive parenting style as perceived parental emotional warmth, because this dimension is the only one in the S-EMBU that measures positive parenting behaviors, and it best matches the core meaning of positive parenting style. The Rejection and Overprotection dimensions reflect negative aspects of parenting and were not within the scope of this study. This subscale uses a 4-point Likert scale (1 = never, 4 = always), with higher scores indicating higher levels of perceived parental emotional warmth. The Cronbach’s alpha coefficient for this subscale in the present study was 0.837. The Chinese version of the S-EMBU (s-EMBU-C) has been validated among Chinese college students, demonstrating good reliability and validity (Jiang et al., 2010).

#### 3.3.2 Physical activity scale

This study used the Physical Activity Rating Scale (PARS-3) revised by Liang Deqing to measure participants’ exercise status over the past month (Deqing, 1994). The scale includes three factors: physical activity intensity, physical activity duration, and physical activity frequency. Physical activity intensity and frequency are scored on a 5-point scale, while physical activity duration is scored on a 4-point scale. The total physical activity score was calculated as intensity × duration × frequency, representing the overall volume of physical activity over the past month. Higher total scores indicate a greater amount of physical activity. The Cronbach’s alpha coefficient for this scale in the present study was 0.951.

#### 3.3.3 Smartphone addiction scale

Smartphone addiction was measured using the Smartphone Addiction Scale Short Version (SAS-SV) developed by Kwon et al. (2013). This scale is unidimensional and includes 10 items (e.g., “I have delayed planned study or work due to smartphone use,” “I have had difficulty concentrating on study or work due to smartphone use”). Each item is rated on a 6-point Likert scale (1 = strongly disagree, 6 = strongly agree), with higher scores indicating more severe smartphone addiction. The Cronbach’s alpha coefficient for this scale in the present study was 0.923. The Chinese version of the SAS-SV has been validated among Chinese college students, showing good psychometric properties (Zhao et al., 2022).

#### 3.3.4 Academic burnout scale

Academic burnout was measured using the Adolescent Learning Burnout Inventory, which was adapted from the Maslach Burnout Inventory (Schaufeli et al., 2002). This scale includes 16 items across three dimensions: physical and emotional exhaustion (4 items, e.g., “I have been feeling empty lately and do not know what to do”), academic cynicism (5 items, e.g., “I feel that I do not understand anyway, so it does not matter whether I study or not”), and reduced personal accomplishment (7 items, e.g., “I can handle exams well”). Each item is rated on a 5-point Likert scale (1 = very true, 5 = very false). Items in the reduced personal accomplishment dimension were reverse coded before analysis so that all items were oriented in the same direction. Higher total scores indicate more severe academic burnout. The Cronbach’s alpha coefficient for this scale in the present study was 0.834. The Chinese version of the ALBI (also referred to as the Adolescent Student Burnout Inventory, ASBI) has been validated among Chinese adolescent and college student populations (Wu et al., 2010).

### 3.4 Data analysis

The data analysis procedure consisted of three steps. There were no missing data in the final analytical sample, as the online questionnaire platform required responses to all items before submission.

First, common method bias was assessed using Harman’s single factor test. An unrotated principal component factor analysis was performed on all items of all scales, and the percentage of variance explained by the first factor was examined to see whether it was below the critical threshold of 40%.

Second, descriptive statistics and correlation analysis were conducted. Means, standard deviations, and Pearson correlation coefficients were calculated for all variables (perceived parental emotional warmth, physical activity, smartphone addiction, academic burnout).

Third, chain mediation analysis was performed using Model 6 (the chain mediation model) in the PROCESS macro (Version 3.4) developed by Hayes (2017). In this model, perceived parental emotional warmth was the independent variable (X), academic burnout was the dependent variable (Y), and physical activity (M1) and smartphone addiction (M2) were the parallel and chain mediators. Gender and grade were included as covariates (household registration type showed no significant correlation with the main variables in preliminary analyses and was therefore not included in the final model). Bias corrected bootstrap method with 5,000 resamples was used to calculate the 95% confidence intervals for the indirect effects. If a confidence interval did not contain zero, the indirect effect was considered statistically significant at the α = 0.05 level. Standardized effect sizes and the percentages of relative mediation were also reported for each pathway.

It should be noted that cross sectional data cannot support causal inference. All statements regarding mediation effects and statistical associations in this study are based on statistical associations and theoretical assumptions. The temporal order among the variables was predetermined by the theoretical model rather than demonstrated by the data themselves.

Prior to the mediation analysis, all key assumptions for multiple regression were checked, including linearity, normality of residuals, homoscedasticity, and influential outliers; all assumptions were met.

## 4 Results

### 4.1 Common method bias test

Because the questionnaire data were obtained through self-reports from participants, Harman’s single factor test was used to examine potential common method bias. An exploratory factor analysis was performed on all items of the Positive Parenting Style Scale, Physical Activity Scale, Smartphone Addiction Scale, and Academic Burnout Scale. Principal component analysis was used to extract common factors, and the first unrotated factor was examined. The results showed that the first factor explained 30.392% of the variance, which is below the critical threshold of 40%. This indicates that no significant common method bias was present in this study.

### 4.2 Descriptive statistics and correlations

The means, standard deviations, and Pearson correlation coefficients for all variables are shown in Table 1. Perceived parental emotional warmth was negatively correlated with academic burnout (*r* = −0.485, *p* < 0.01). Perceived parental emotional warmth was positively correlated with physical activity (*r* = 0.450, *p* < 0.01) and negatively correlated with smartphone addiction (*r* = −0.357, *p* < 0.01). Physical activity was negatively correlated with smartphone addiction (*r* = −0.615, *p* < 0.01) and with academic burnout (*r* = −0.459, *p* < 0.01). Smartphone addiction was positively correlated with academic burnout (*r* = 0.689, *p* < 0.01).

**TABLE 1:** Descriptive statistics and correlation analysis of variables.

Variable	M	SD	Perceived parental emotional warmth	Physical activity	Smartphone addiction	Academic burnout
Perceived parental emotional warmth	22.910	1.380	1	1	1	1
Physical activity	74.93	29.627	0.450**			
Smartphone addiction	38.38	10.955	−0.357**	−0.615**		
Academic burnout	49.940	9.600	−0.485**	−0.459**	0.689**	

**p < 0.01.

To assess whether the four constructs were empirically distinct, we conducted *post-hoc* discriminant validity analyses. The square root of the average variance extracted (√AVE) for perceived parental emotional warmth was 0.251, for physical activity 0.956, for smartphone addiction 0.739, and for academic burnout 0.488. When compared against the inter-construct correlations, several pairs showed inadequate discriminant validity. Notably, the correlation between academic burnout and physical activity (*r* = 0.848) substantially exceeded the √AVE of academic burnout (0.488), and the correlation between perceived parental emotional warmth and academic burnout (*r* = 0.485) exceeded the √AVE of perceived parental emotional warmth (0.251). These results indicate substantial construct overlap, particularly involving the academic burnout measure. Consequently, the high *R*^2^ value (0.772) reported in the combined regression model should be interpreted with caution, as it may partly reflect shared measurement variance rather than purely substantive relationships.

### 4.3 Perceived parental emotional warmth and academic burnout among university students: chain-mediated effects

To examine the bivariate statistical associations between each predictor and academic burnout, three separate simple regression models were estimated, with perceived parental emotional warmth, physical activity, and smartphone addiction entered as independent variables in each respective model and academic burnout as the dependent variable (Table 2). The results showed that perceived parental emotional warmth showed a negative statistical association with academic burnout (β = −0.485, *t* = −19.109, *p* < 0.001). Physical activity showed a negative statistical association with academic burnout (β = −0.459, *t* = −55.087, *p* < 0.001). Smartphone addiction showed a positive statistical association with academic burnout (β = 0.604, *t* = 32.794, *p* < 0.001).

**TABLE 2:** Regression analysis of perceived parental emotional warmth, physical activity, smartphone addiction, and academic burnout.

Variable	Academic burnout			
	β	*T*	F	R^2^
Perceived parental emotional warmth	−0.485	−19.109***	365.160	0.234
Physical activity	−0.459	−55.087***	3,034.591	0.718
Smartphone addiction	0.604	32.794***	1,075.473	0.474

***p < 0.001.

Using perceived parental emotional warmth (X) as the independent variable, academic burnout (Y) as the dependent variable, and physical activity (W1) and smartphone addiction (W2) as mediators, this study tested the chain mediation effect of physical activity and smartphone addiction on the relationship between perceived parental emotional warmth and academic burnout, after controlling for demographic variables (gender and grade). The analysis was conducted using the PROCESS macro (Version 3.4) in SPSS 25.0, applying Model 6 to test the chain mediation hypothesis. The significance of the indirect effects was examined using the bootstrap method with 5,000 resamples and 95% confidence intervals (CIs).

As shown in Table 3, perceived parental emotional warmth showed a significant negative statistical association with academic burnout (β = −0.485, *p* < 0.001, 95% CI [−0.534, −0.435]). This indicates that a higher score on perceived parental emotional warmth is associated with a lower level of academic burnout, supporting Hypothesis 1. The relationship between perceived parental emotional warmth and academic burnout was mediated by physical activity (β = −0.290, 95% CI [−0.323, −0.257]), suggesting that higher perceived parental emotional warmth is associated with lower academic burnout through increased physical activity, thus supporting Hypothesis 2. Smartphone addiction also mediated the relationship between perceived parental emotional warmth and academic burnout (β = −0.026, 95% CI [−0.040, −0.012]), indicating that perceived parental emotional warmth is associated with lower academic burnout through reduced smartphone addiction, supporting Hypothesis 3. Finally, physical activity and smartphone addiction sequentially mediated the relationship (β = −0.066, 95% CI [−0.080, −0.054]), demonstrating that perceived parental emotional warmth is associated with lower academic burnout through the chain pathway of increased physical activity and then reduced smartphone addiction, thereby supporting Hypothesis 4.

**TABLE 3:** Mediation effect values and effect sizes.

Path	Effect	BOOT SE	BOOT LLCI	BOOT ULCI	Relative mediation effect
Total effect	−0.485***	0.025	−0.534	−0.435	100%
Direct effect	−0.104***	0.016	−0.134	−0.073	21.443%
Total indirect effect	−0.381***	0.018	−0.418	−0.346	78.557%
Indirect effect 1 (physical activity)	−0.290***	0.017	−0.323	−0.257	59.794%
Indirect effect 2 (smartphone addiction)	−0.026***	0.007	−0.040	−0.012	5.361%
Indirect effect 3 (physical activity and smartphone addiction)	−0.066***	0.007	−0.080	−0.054	13.608%

Indirect effect 1: perceived parental emotional warmth → physical activity → academic burnout; Indirect effect 2: perceived parental emotional warmth → smartphone addiction → academic burnout; Indirect effect 3: perceived parental emotional warmth → physical activity → smartphone addiction → academic burnout. ***p < 0.001, All reported effects are completely standardized coefficients (β).

**FIGURE 2:**
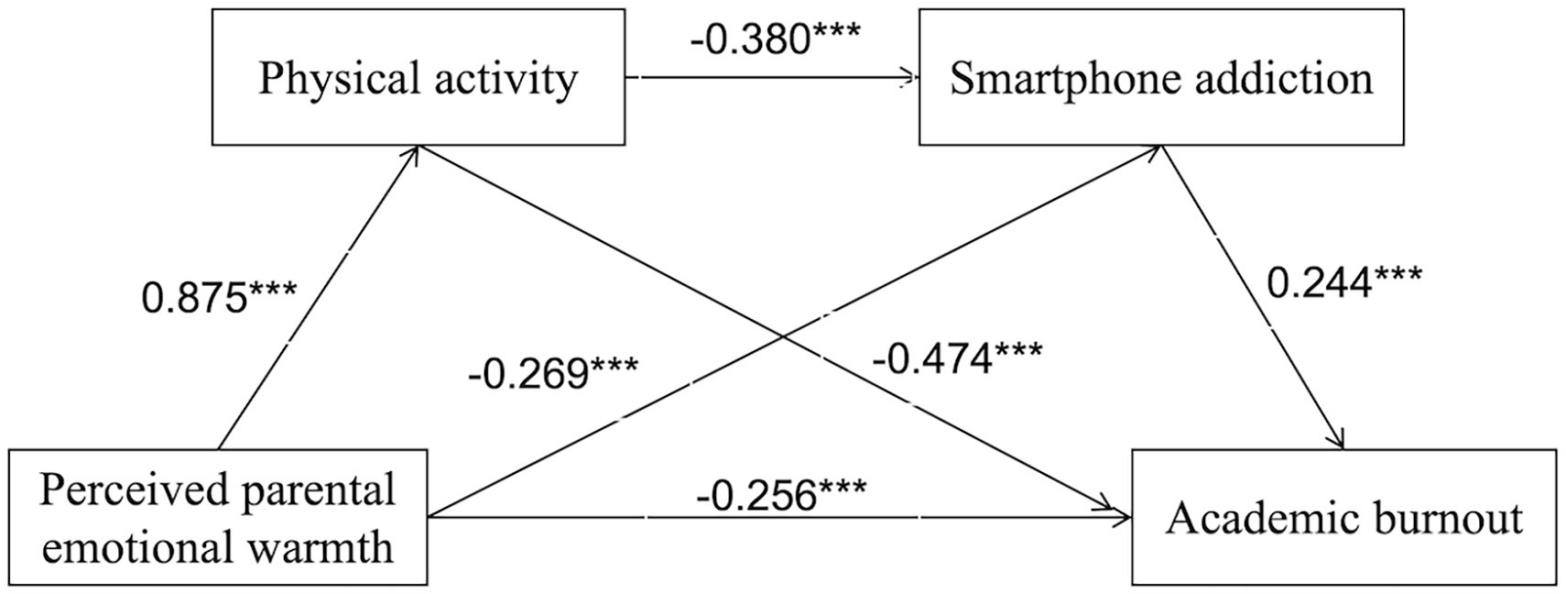
Model of chain mediating roles of physical activity and smartphone addiction between perceived parental emotional warmth and academic burnout. ***p < 0.001 (statistically highly significant).

In addition, the effect sizes of all three indirect pathways and the total effect were statistically significant (*p* < 0.001). The total effect was −0.485, with a direct effect of −0.104 (accounting for 21.443% of the total effect) and a total indirect effect of −0.381 (accounting for 78.557%). For the indirect pathways: Path 1 (perceived parental emotional warmth → physical activity → academic burnout) had an effect size of −0.290 (59.794% of the total indirect effect); Path 2 (perceived parental emotional warmth → smartphone addiction → academic burnout) had an effect size of −0.026 (5.361% of the total indirect effect); Path 3 (perceived parental emotional warmth → physical activity → smartphone addiction → academic burnout) had an effect size of −0.066 (13.608% of the total indirect effect). The specific pathways are shown in Figure 2.

To examine the unique statistical associations of all predictors simultaneously, a multiple regression model was estimated with perceived parental emotional warmth, physical activity, and smartphone addiction entered together as predictors of academic burnout (Table 4). This combined model complements the bivariate analyses presented in Table 2 by showing each variable’s contribution while controlling for the others. Multicollinearity diagnostics were performed for the independent variables in the regression model (Table 4). The variance inflation factor (VIF) values were 1.27 for perceived parental emotional warmth, 1.78 for physical activity, and 1.63 for smartphone addiction. All VIF values were below the commonly used threshold of 5, indicating that no serious multicollinearity was present among the independent variables. Although physical activity and smartphone addiction were moderately correlated (*r* = −0.615), the VIF values were well below the strict threshold of 10, so the impact of collinearity on the estimation of regression coefficients was negligible.

**TABLE 4:** Linear regression analysis results.

Variable	Unstandardized coefficients		Standardized coefficients	*t*	*p*	Collinearity statistics	
	B	Std. error	Beta			VIF	Tolerance
Constant	73.429	2.587	–	28.384	0.000	–	–
Perceived parental emotional warmth	−0.721	0.109	−0.104	−6.633	0.000	1.270	0.787
Physical activity	−0.208	0.006	−0.643	−34.776	0.000	1.783	0.561
Smartphone addiction	0.225	0.015	0.257	14.518	0.000	1.629	0.614
R2	0.772						
R2adj	0.772						
F	1,341.486***						
D-W	2.001						

***p < 0.001.

The *R*^2^ value of 0.772 indicates that the three predictors together explain a large proportion of the variance in academic burnout. This is largely driven by the strong bivariate correlation between smartphone addiction and academic burnout (*r* = 0.689). However, as noted in the discriminant validity assessment (Section “4.2 Descriptive statistics and correlations”), the academic burnout measure exhibited considerable construct overlap with both perceived parental emotional warmth and physical activity in this sample. Therefore, while the VIF values (all < 1.8) confirm that multicollinearity did not inflate the explained variance, this high *R*^2^ should still be interpreted with caution, as it may partially reflect shared measurement variance rather than purely substantive relationships.

## 5 Discussion

Based on self-determination theory and the behavioral substitution perspective, this study aimed to examine the statistical associations among perceived parental emotional warmth, physical activity, smartphone addiction, and academic burnout in physical education pre-service teachers, as well as their possible sequential patterns. The cross sectional data analysis showed that perceived parental emotional warmth was not only directly associated with academic burnout but also linked through a statistical pattern consistent with a chain mediation (physical activity → smartphone addiction). This pattern suggests that perceived parental emotional warmth may be associated with higher levels of physical activity in individuals, which in turn is associated with lower smartphone addiction tendency, and ultimately with lower levels of academic burnout. The identification of this statistical pattern offers an integrated theoretical framework for understanding the potential processes linking family environment and academic adaptation from the perspective of behavioral resource competition.

### 5.1 Direct association of perceived parental emotional warmth

The results of this study showed a significant negative association between perceived parental emotional warmth and academic burnout. This finding is consistent with existing literature showing that a warm family emotional environment, as a key adaptive resource, is negatively associated with academic burnout (Shin et al., 2012, Zhang et al., 2023). Specifically, using the measure employed in this study, higher scores on perceived parental emotional warmth typically indicate that children perceive higher levels of emotional warmth and understanding, as well as lower levels of rejection, denial, and overprotection. Such a warm and supportive parental emotional climate can satisfy individuals’ basic psychological needs for autonomy, competence, and relatedness (Slemp et al., 2024). For physical education pre-service teachers, the dual roles of academic study and professional training may make them particularly sensitive to family emotional support and autonomous space. Therefore, higher levels of perceived parental emotional warmth may provide a more stable and nourishing foundation of psychological resources to cope with dual challenges and thus be associated with lower levels of academic burnout. This finding confirms that perceived parental emotional warmth is an important correlate of academic psychological adaptation among physical education pre-service teachers.

### 5.2 Mediating role of physical activity

This study further revealed the key mediating role of physical activity. This finding is consistent with the classic theoretical model of “family support → health behavior → mental health” and provides empirical support for it in the domain of academic burnout (Yang et al., 2026). According to self-determination theory, the emotional support and autonomy climate reflected by a perceived parental emotional warmth is theoretically linked to children’s intrinsic motivation, which may be associated with the adoption of regular physical activity as a habit of autonomous choice and enjoyment rather than an externally imposed task (Sebire et al., 2013). Especially for physical education pre-service teachers, physical activity is deeply embedded in their professional identity and daily routines, and this association may be strengthened, so that positive influences from the family may be more directly reflected in behavioral persistence in maintaining systematic training. More importantly, regular physical activity is statistically associated with a range of physiological and psychological benefits—such as increased vitality, improved sleep (Ye et al., 2023), enhanced self-efficacy through skill mastery (Netz et al., 2005), and positive emotions that counteract stress—which in turn are associated with lower academic burnout (Wang et al., 2025a). These physical and mental resources associated with exercise may serve as a buffer against academic emotional exhaustion and cynicism. From a life-course perspective, continuity in sport participation across developmental stages—from childhood through adolescence into adulthood may shape future teachers’ physical activity habits and professional attitudes (Daga et al., 2026). This suggests that the mediating role of physical activity identified in this study may be understood in light of a long-term developmental trajectory rather than reflecting only current behavioral choices. Therefore, this pathway not only suggests the generality of physical activity as a mediator but also points to its importance as a profession specific protective factor among students with high physical demands.

### 5.3 Mediating role of smartphone addiction

This study found that smartphone addiction is another important mediating pathway linking perceived parental emotional warmth and academic burnout. It should be noted that our focus is specifically on problematic smartphone use characterized by loss of control and negative consequences, rather than functional or academic use of smartphones. This finding provides a family-based perspective for understanding risk factors of academic burnout in the digital age. The underlying mechanism can be explained by compensatory use theory. When individuals perceive low levels of perceived parental emotional warmth, which may indicate a lack of emotional warmth or strong overcontrol, their basic psychological needs for autonomy and relatedness are not sufficiently satisfied in real family interactions. To compensate for this lack of basic psychological needs, individuals may turn to the virtual environment provided by smartphones, seeking immediate social connections, entertainment feedback, and a sense of control. If this pattern persists over time, it may easily be associated with addiction (Panova and Carbonell, 2018). Smartphone addiction is theoretically associated with poorer academic adaptation through multiple mechanisms. First, it excessively occupies and fragments core study time, which is associated with scattered attention (Im and Jang, 2017). Second, it depletes cognitive and self-regulatory resources, which is associated with a greater likelihood to give up when facing academic difficulties (Al-Abyadh et al., 2024). Third, it disrupts sleep rhythms and affects daytime energy and cognitive function (AydıN, 2025). Fourth, it may be associated with weaker real life interpersonal interactions and exacerbate academic cynicism (Kraut et al., 1998). The results of this study are consistent with recent research that views smartphone addiction as a key proximal correlate for academic burnout (Paterna et al., 2024), but they further point to perceived parental emotional warmth as a related factor. This suggests that efforts to address student smartphone addiction should not be limited to behavioral control alone; attention should also be paid to improving the family support system and the satisfaction of psychological needs behind it.

The strong correlation between smartphone addiction and academic burnout (*r* = 0.689) warrants comment. Although both constructs are conceptually distinct, with one reflecting problematic technology use and the other an academic stress-related syndrome, the high correlation suggests some shared variance. The VIF values (Table 4) indicate no multicollinearity issue, supporting their statistical distinctiveness. However, the *post-hoc* discriminant validity assessment (Section “4.2 Descriptive statistics and correlations”) revealed that the more critical concern involves the academic burnout construct’s overlap with physical activity (*r* = 0.848) and, to a lesser extent, with perceived parental emotional warmth. This measurement limitation suggests that the observed associations, particularly those involving physical activity, should be interpreted with appropriate caution.

### 5.4 Chain mediating role

The core theoretical contribution of this study is the identification, based on cross sectional data, of a statistical pattern consistent with the chain sequence of “perceived parental emotional warmth → physical activity → smartphone addiction → academic burnout.” This pattern should be interpreted as a theoretically plausible sequence requiring confirmation in future longitudinal research. This pattern suggests that perceived parental emotional warmth is statistically associated with a sequence linking constructive behaviors to risky behaviors, which in turn is associated with academic adaptation. Specifically, higher perceived parental emotional warmth is associated with higher levels of physical activity, and higher physical activity is associated with lower smartphone addiction tendency, which in turn is associated with lower levels of academic burnout.

A perceived parental emotional warmth provides individuals with an emotional environment that satisfies basic psychological needs, which first is theoretically linked to the formation and maintenance of physical activity as a constructive health habit. Regular physical activity is not only associated with better physical and mental health, but more importantly, it may be associated with lower susceptibility to smartphone addiction through a logic of behavioral competition and substitution. Physical activity occupies time, but more importantly, it is associated with positive emotional experiences, abundant energy, and confidence in one’s own abilities. The enhancement of these internal resources is associated with a lower need for individuals to rely excessively on the virtual world to compensate for psychological deficiencies or escape from stress, which may in turn be associated with lower motivation for problematic smartphone use. At the same time, the time and cognitive resources invested in physical activity may also be associated with fewer opportunities that might otherwise be used for excessive smartphone use. Thus, physical activity shows a direct negative statistical association with academic burnout, and is also indirectly associated with reduced risky behaviors (smartphone addiction) in the sequential model, forming a coherent chain of “health promotion and risk inhibition.”

The statistical support for this chain pathway represents a valuable theoretical integration. It connects self-determination theory, which starts from psychological need satisfaction, with the behavioral substitution perspective, which emphasizes dynamic relationships between behaviors. Self-determination theory explains the initial link of the chain: how a supportive family environment is theoretically linked to intrinsic motivation and health behaviors. The behavioral substitution perspective clearly explains the subsequent link: how, given the finite time and attention resources of an individual, the investment in one constructive activity may compete with the time and resources available for another consumptive activity. This integrated model suggests that for physical education pre-service teachers, encouraging physical activity is not only about adding a healthy activity but also about supporting a positive pattern that may be associated with better daily behavioral structure and actively manages digital life, offering a theoretical basis for considering preventive strategies.

Regarding the potential mechanisms underlying the negative association between physical activity and smartphone addiction, at least three complementary perspectives can be proposed, though these mechanisms were not directly tested in the present study. First, competition for time and cognitive resources: regular physical activity and smartphone use may compete for limited resources such as time and attention, and higher levels of physical activity may be associated with less screen time (Pirwani and Szabo, 2025). Second, emotional substitution effect: physical activity is associated with positive emotional states, and this positive emotional experience from exercise may be associated with a reduced motivation to seek immediate emotional compensation through smartphones (Shi et al., 2025). Third, transfer of self-control ability: long term adherence to physical activity may require self-discipline and delayed gratification, and these abilities may be positively associated with inhibitory control over smartphone use (Şwia̧tek et al., 2023). It should be emphasized that the above mechanisms are speculations based on existing theories; the data from this study cannot distinguish the specific pathways of these mechanisms, nor can they rule out other explanations.

It is worth noting that the chain mediation mechanism described above may have some specificity in the group of physical education pre-service teachers. First, for this group, physical activity goes beyond general health behavior and is deeply embedded in their professional identity and daily training regimen. The emotional support and autonomous space provided by perceived parental emotional warmth may be more directly reflected in adherence to systematic training plans rather than generalized exercise willingness. This suggests that the effect size of the “parenting → physical activity” pathway in this study may differ from that in general university student populations. Second, physical education pre-service teachers simultaneously bear the cognitive pressure of academic study and the load of physical training. The consumption of physical and mental resources under this dual pressure is more intense, which makes them both prone to using smartphones as a quick “shortcut” for emotional recovery (thus increasing the risk of addiction) and also makes the marginal benefit of regular physical activity as a resource recovery pathway more prominent. In other words, the negative association in the chain mediation of “physical activity → smartphone addiction” may be more pronounced in this group. Finally, the professional identity bound to physical activity also means that the improvements in self-efficacy and sense of accomplishment associated with exercise may be associated with lower levels of general academic burnout but may also be associated with lower levels of profession related burnout components caused by failure to meet technical standards or competition losses. Therefore, the chain pathway revealed in this study may be related to both behavioral ecological regulation and professional identity maintenance among physical education pre-service teachers.

### 5.5 Theoretical contributions and practical implications

The findings of this study offer preliminary insights for understanding and supporting the academic and psychological development of physical education pre-service teachers. The patterns of associations revealed by the cross-sectional data suggest that future support efforts could adopt an integrated ecological perspective, focusing on the continuous process from family environment to individual behaviors.

Perceived parental emotional warmth showed close associations with academic burnout and health behaviors. This highlights the potential importance of family-related emotional factors in understanding student wellbeing, though the present data do not provide a basis for recommending family-based efforts.

Meanwhile, the central position of physical activity in the association model deserves further attention and application. For physical education pre-service teachers, physical activity has the dual attribute of being both a professional skill and a protective factor for mental health. Systematically integrating knowledge of sport psychology into professional training and creating a supportive campus sports atmosphere may support students in adopting regular exercise as an effective self-regulation tool, which may be associated with more proactive management of academic stress.

Regarding digital behavior guidance, this study revealed a possible trade off relationship between physical activity and smartphone use. This suggests that the focus of future efforts could shift from simple “use restriction” to “enriching real life experiences.” By providing attractive offline activities, especially sports participation and team collaboration that offer immediate positive feedback, students’ real-life experiences can be effectively enriched, which may in turn be associated with a reduced need for compensatory reliance on virtual spaces and with more balanced digital habits.

These directional implications based on the current findings await verification by future research using longitudinal follow up or intervention experiments. Given the cross-sectional nature of this study, these practical implications should be interpreted as preliminary and hypothesis-generating rather than evidence-based recommendations. Exploring more complex mechanisms and individual differences will help develop more targeted and effective comprehensive support strategies, thereby bridging theoretical associations with practical applications.

### 5.6 Limitations

This study preliminarily examined the chain mediating mechanism through which perceived parental emotional warmth is associated with academic burnout via physical activity and smartphone addiction. However, several limitations should be addressed in future research.

First, a limitation of the study design. This study used a cross-sectional design, with all variables collected at the same time point. Although we proposed a temporal order among the variables based on theory (perceived parental emotional warmth → physical activity → smartphone addiction → academic burnout), cross sectional data cannot establish causal relationships or rule out the possibility of reverse causation. For example, high academic burnout might lead individuals to reduce physical activity and rely more on smartphones as an emotional escape tool; smartphone addiction might also reduce physical activity participation. In addition, bidirectional reinforcing relationships may exist among the variables (e.g., smartphone addiction and academic burnout may exacerbate each other). Future research could use multi time point longitudinal designs (such as cross lagged analysis) or randomized controlled experiments to clarify the temporal relationships among the variables.

Second, limitations regarding sample representativeness and response bias. The effective response rate in this study was 91.69% (1,192/1,300). The 108 nonresponsive questionnaires were mainly excluded due to abnormal completion times or invalid response patterns. Because the data were collected anonymously online, we were unable to compare the differences in demographic characteristics or core variables between responders and non-responders (i.e., those excluded). Although the high response rate reduced the risk of non-response bias to some extent, it cannot be completely ruled out. Moreover, the sample was limited to Chinese physical education pre-service teachers, so the generalizability of the conclusions to other cultural backgrounds, other majors, or middle school student populations needs further verification.

Third, limitations of measurement methods. All data came from participants’ self-reports, which might introduce social desirability bias. This is particularly relevant when reporting parenting style and smartphone addiction, as participants might tend to provide socially expected answers. Although we incorporated fixed-response items to reduce inattentive responding and Harman’s single-factor test indicated no severe common method bias, this test is not sufficient to completely rule out common method variance. Future research could introduce multi-source data or objective measurements to improve objectivity.

Fourth, limitations in mechanism testing. This study verified the chain mediation effects of physical activity and smartphone addiction but did not test why this chain pathway holds. The mechanisms proposed in the discussion, such as resource competition, emotional substitution, and transfer of self-control, are speculations based on theoretical literature. The empirical data of this study cannot distinguish which mechanism plays a dominant role, nor can they rule out the possibility that unmeasured third variables (e.g., conscientiousness as a personality trait, time management ability, family socioeconomic status) simultaneously affect multiple variables. Future research could use ecological momentary assessment (EMA) to capture the dynamic competitive relationship between physical activity and smartphone use in real contexts, and measure potential mediating variables (e.g., emotional state, self-control resources) to test specific mechanisms.

Fifth, limitations in model simplification and extension. The chain mediation model proposed in this study included only two mediators, but the factors influencing academic burnout are more complex. For example, psychological resilience, academic self-efficacy, and peer support may play additional mediating or moderating roles in the model. In addition, the relationship between physical activity and smartphone use may vary by activity type (team sports vs. individual sports), purpose of use (social, entertainment, or study), and family socioeconomic status. Future research could construct more complex integrated models (e.g., moderated mediation models) and explore other potential theoretical pathways.

Sixth, although we argued that physical education pre-service teachers represent a particularly relevant population, our single-group design did not include a comparison group. The specificity of the observed pathways to this population therefore remains speculative. Cross-group comparisons are needed to test whether these mediating effects are unique to PE pre-service teachers or generalizable to other student populations.

Seventh, the proposed model may have suffered from omitted variable bias. Factors such as personality traits, academic self-efficacy, resilience, and socioeconomic status were not included, yet they may simultaneously affect physical activity, smartphone addiction, and academic burnout. Future research incorporating these covariates is warranted to test the robustness of the observed associations.

Eighth, a further methodological limitation concerns discriminant validity. Post-hoc analyses revealed that the square root of the AVE for academic burnout (0.488) was substantially lower than its correlation with physical activity (*r* = 0.848), and the √AVE for perceived parental emotional warmth (0.251) was lower than its correlation with academic burnout (*r* = 0.485). This indicates considerable construct overlap in this sample. A plausible explanation lies in the nature of the population: physical education pre-service teachers experience high levels of physical fatigue from daily training, and the ALBI’s “physical and emotional exhaustion” dimension may tap into this training-induced fatigue rather than purely academic burnout. Consequently, the empirical distinction between “academic exhaustion” and “physical activity load” becomes blurred. This measurement confounding suggests that the mediation effects, particularly those involving physical activity, may be inflated. Future studies should employ refined measurement instruments that more sharply differentiate academic burnout from health behaviors and physical fatigue.

Ninth, the exclusion of participants with diagnosed mental illness or recent psychological treatment may have reduced confounding, but could also have restricted the range of academic burnout scores and potentially underestimated the true associations. Future studies including a broader clinical spectrum are warranted to examine whether the proposed mediation model holds across different levels of psychological functioning.

## 6 Conclusion

Based on self-determination theory and the behavioral substitution perspective, this study examined the statistical associations among perceived parental emotional warmth, physical activity, smartphone addiction, and academic burnout in physical education pre-service teachers. Analyzing cross-sectional data from 1,192 participants, the findings show that perceived parental emotional warmth is associated with lower academic burnout both directly and indirectly through three pathways: via physical activity alone, via smartphone addiction alone, and sequentially via physical activity followed by smartphone addiction.

These findings offer a behavioral-ecological perspective on how perceived parental emotional warmth may relate to academic adaptation through a sequence of constructive behaviors (physical activity) and reduced risky behaviors (smartphone addiction). However, given the cross-sectional design, causal inferences cannot be drawn, and the mediation should be interpreted as statistical associations consistent with the theoretical model rather than evidence of causal processes.

The practical implications are preliminary and hypothesis-generating rather than evidence-based recommendations. They tentatively suggest that supporting physical activity and enriching offline experiences may be relevant considerations for future support strategies, though such applications require confirmation through longitudinal or experimental research.

Future longitudinal and experimental studies are critically needed to confirm the temporal ordering of the proposed pathways and to test whether interventions aimed at increasing physical activity can effectively reduce problematic smartphone use and academic burnout. Such research would help translate these preliminary associations into evidence-based strategies for supporting the wellbeing of physical education pre-service teachers. Critically, future research should also employ measurement instruments with stronger discriminant validity to ensure that observed associations reflect genuine construct-level relationships rather than measurement content overlap.

## Data Availability

The original contributions presented in this study are included in this article/Supplementary material, further inquiries can be directed to the corresponding author.

## Footnotes

1
https://www.wenjuan.com/s/UZBZJvYWmq/#
